# Case Report: Deutetrabenazine as an Adjunctive Treatment for Stuttering

**DOI:** 10.3389/fpsyt.2021.683368

**Published:** 2021-10-25

**Authors:** Catherine A. Ha, Gerald A. Maguire

**Affiliations:** ^1^School of Medicine, University of California, Riverside, Riverside, CA, United States; ^2^Department of Psychiatry and Neuroscience, University of California, Riverside, Riverside, CA, United States

**Keywords:** stuttering, deutetrabenazine, VMAT 2, D2 receptor antagonists, tardive dyskinesia (TD)

## Abstract

Childhood-Onset Fluency Disorder (Stuttering) is a neurodevelopmental disorder in which disturbances occur in the normal fluency and time patterning of speech. While the dopamine system has been well-described in its neurophysiology, there currently is no FDA-approved treatment for stuttering. Second-generation antipsychotics, which have been effective in the treatment of schizophrenia and bipolar disorder, act as dopamine D-2 receptor antagonists at the postsynaptic neuron and have been shown to reduce the symptoms of stuttering. However, the D-2 receptor antagonist and partial agonist agents carry the potential for metabolic side effects and can potentially lead to movement disorders. Deutetrabenazine, a VMAT-2 inhibitor indicated to treat hyperkinetic movement disorders, is a potential candidate in the treatment of stuttering, based on its mechanism of action in decreasing dopamine activity while not carrying the risk of metabolic adverse events.

## Introduction

Stuttering is a neurodevelopmental disorder that affects 1% of the adult population in an ~4:1 male to female ratio (1, 2). Current research on stuttering reflects a multifactorial process with physiologic etiology. Neurophysiology studies demonstrate the role of the dopamine system in the pathogenesis of stuttering, with an improvement of symptoms from treatment with dopamine D-2 receptor antagonists (3–6).

Second-generation antipsychotics act on the dopamine D-2 receptor as either antagonists or partial agonists and have been effective in the treatment of not only schizophrenia and bipolar disorder, but also have been shown, in limited trials, to improve symptoms of stuttering (7–9). In both first- and second-generation antipsychotics, there is a risk of developing drug induced movement disorders (D-IMDs), a spectrum of neurologic motor disturbances characterized by abnormally increased or decreased motor activity and function (10). D-IMDs can be distinguished into reversible or persistent, hypokinetic or hyperkinetic, and dystonic or non-dystonic classifications (10). This constellation of presentations includes acute dystonia, drug induced parkinsonism, and bradykinesia, and can precede the eventual development of the overreactive dopamine state known as tardive dyskinesia (TD). TD is a medication-induced hyperkinetic movement disorder caused by exposure to dopamine receptor-blocking agents that persists for at least 1 month after discontinuation of the offending agent (1). In comparison to other D-IMDs that present more acutely, TD is distinguished by its later, insidious onset. While the repetitive, involuntary movements may manifest as a spectrum of orofacial dyskinesia, dystonia, chorea, and tics, TD most commonly presents with oro-bucco-lingual and facial dyskinesias characterized by protruding and twisting movements of the tongue, chewing movements, blepharospasm, and pouting, puckering, or smacking movements of the lips (11).

Although the rates of D-IMDs and TD are less in second generation antipsychotic agents, such can still occur. These agents may also be associated with the potential of metabolic adverse events, but overall tend to be well-tolerated in most subjects (12). The dopamine acting agents have been shown in several studies, although many limited in scope, to be effective in the treatment of developmental stuttering. However, the studies demonstrate that not all patients respond to D-2 agents and the search for alternative mechanistic agents is warranted. A class of medications, the VMAT-2 inhibitors, are approved to treat TD in the United States, and as this case describes, may potentially serve as an adjunctive treatment in stuttering as well. The VMAT-2 inhibitors decrease dopamine activity not by antagonizing the receptor, but rather by decreasing dopamine levels in the presynaptic neuron. While VMAT-2 inhibitors predominantly decrease dopamine activity, depending on their specific compound, they may also decrease the activity of other monoamine neurotransmitters and may potentially lead to depressive symptoms.

## Case Description

A healthy 20-year-old Caucasian male with no family history presented with a history of stuttering at age 6 years. The patient had attended an annual 1-week intensive residential speech therapy program for eight consecutive years and continued speech therapy but experienced a poor response.

At age 14, patient sought care for his stuttering due to lack of efficacy from his speech therapy. Aripiprazole, 5 mg per day, was initiated and titrated to 30 mg per day based on his tolerability, to which the patient exhibited a positive response (1). He subsequently developed a thirty-pound weight gain over the course of 5 years above his natural growth. The patient was then switched to lurasidone 60 mg per day for 1 year and experienced a thirty-pound weight loss but worsening of his speech to pre-treatment level. Patient requested to return to aripiprazole and was restarted on 30 mg per day with a resultant improvement of his speech again. Topiramate was initiated as aripiprazole was started to prevent weight gain and was proven successful. However, after 6 months of receiving aripiprazole, the patient developed minor involuntary movements of his tongue and was diagnosed as having tardive dyskinesia Table 1.

**Table 1:** Timeline.

**Month**	**Presentation**	**Treatments**	**Height (cm)**	**Weight (kg)**	**BMI**	**AIMS**
1	Stuttering, non-responsive to speech therapy.	Started on aripiprazole eventually titrated to 30 mg per day.	165.74 cm	60.78 kg	22.24 kg/m^2^	0
3	Stuttering stable.	Continued on aripiprazole 30 mg per day.	168.91 cm	73.03 kg	25.6 kg/m^2^	0
15	Weight gain. No tardive dyskinesia.	Aripiprazole 30 mg per day Counseled on diet and exercise.	170.18 cm	86.64 kg	29.95 kg/m^2^	0
20	Evaluated by endocrinology due to BMI > 30.	Aripiprazole 30 mg per day. Initiated diet and exercise program.	170.18 cm	88.90 kg	30.72 kg/m^2^	0
40	Weight gain persisted. No tardive dyskinesia.	Discontinued aripiprazole 30 mg per day. Started lurasidone 20 mg, titrated to 60 mg per day.	170.18 cm	72.57 kg	24.69 kg/m^2^	0
44	Stuttering worsened back to pre-treatment levels.	Increased lurasidone 80 mg per day.	170.18 cm	73.03 kg	25.2 kg/m^2^	0
49	Stuttering non-responsive to lurasidone. Patient noted weight stable	Re-start aripiprazole, titrated to 30 mg per day. Tapered and discontinued lurasidone 80 mg. Topiramate titrated to 100 mg per day to prevent weight gain.	170.18 cm	Unable to obtain	Unable to obtain	0
50	Speech improved with aripiprazole. Patient noted weight stable.	Continued aripiprazole 30 mg per day and topiramate.	170.18 cm	Unable to obtain	Unable to obtain	
51	Tongue dyskinesias noted on exam and subjectively by patient. Patient noted weight stable.	Continued aripiprazole 30 mg per day. Started deutetrabenazine 6 mg per day.	170.18 cm	Unable to obtain	Unable to obtain	7
52	Tongue dyskinesia subjectively improved as noted by patient. Stuttering stable. Patient noted weight stable.	Aripiprazole 30 mg per day. Deutetrabenazine titrated to 9 mg twice per day.	170.18 cm	Unable to obtain	Unable to obtain	5
54	Stuttering stable. Patient noted weight stable.	Aripiprazole 30 mg per day. Deutetrabenazine 12 mg twice per day.	170.18 cm	Unable to obtain	Unable to obtain	2
56	Stuttering stable. Weight stable. No movements elicited on AIMS examination.	Continued aripiprazole 30 mg per day and deutetrabenazine 12 mg twice per day.	170.18 cm	69.85 kg	24.12 kg/m^2^	0

*Certain weight and BMI measurements were unable to be obtained due to the nature of telemedicine visits during the COVID-19 pandemic*.

Deutetrabenazine was initiated at 6 mg per day, and then increased over time to 12 mg twice a day with resultant improvement of his tardive dyskinesia and further improvement of his stuttering from moderate to essentially mild on the Abnormal Involuntary Movements Scale (AIMS). The patient continues to tolerate both deutetrabenazine and aripiprazole well.

## Discussion

Deutetrabenazine, along with valbenazine, which have received FDA approval to treat tardive dyskinesia, may hold promise as potential pharmacologic treatments for stuttering. Such agents, as this case illustrates, may be administered concomitantly with dopamine antagonists and partial agonists to reduce dyskinetic symptoms and possibly enhance the therapy for stuttering. The patient presented in this case tolerated both aripiprazole and deutetrabenazine well, and did not experience any potential adverse events, such as depression, that may be associated with monoamine decrease.

Further research is necessary, however, to determine if such pharmacologic augmentation provides a synergism in therapeutic effect. One issue is more certain—the VMAT-2 inhibitors possess a different side-effect profile than the D-2 antagonists by avoiding metabolic concerns and the risk of long-term movement disorders. However, they themselves are not without risks such as depression, cardiac conduction delays, and muscle stiffness. Additional research with controlled studies to determine possible monotherapy or augmentation therapy of VMAT-2 inhibitors in stuttering may be warranted in the future. Given the short duration of treatment as illustrated by the case, long-term, controlled studies are required to address the potential effect of deutetrabenazine as a monotherapy in the setting of stuttering associated with tardive dyskinesia.

## Patient Perspective

Patient subjectively noted stuttering and tongue dyskinesias. However, the patient wishes to maintain on aripiprazole due to the improvement of his speech, in spite of the tongue dyskinesias and weight gain. Therefore, treatments of these side effects have been initiated and successful.

“Prior to starting Deutetrabenazine, I have been taking 30 mg of aripiprazole daily for stuttering. This has helped with my fluency, but I thought that my fluency could still be better. The aripiprazole was also causing uncontrolled movements in my mouth area (Dyskinesia). I was prescribed deutetrabenazine to treat my stuttering and dyskinesia. My stuttering has improved since I started taking deutetrabenazine. I feel like I am in better control of my speech. I also feel like I can more easily use the “tools” I have previously learned in speech therapy (I am no longer in speech therapy, but I remember the content of what I learned). The dyskinesia has also improved since I started deutetrabenazine; however, I still experience some uncontrolled movements in my mouth.”

## Data Availability Statement

The original contributions presented in the study are included in the article/supplementary material, further inquiries can be directed to the corresponding author/s.

## Author Contributions

GM and CH participated directly in the patient's care. CH wrote the manuscript with support from GM. All authors provided critical feedback and helped shape the research, analysis, and manuscript.

## Conflict of Interest

The University of California, Riverside holds the patent with GM as inventor for VMAT-2 inhibitors in the treatment of stuttering. GM discloses research grants from Emalex and Teva and consulting with Vivera. The remaining author declares that the research was conducted in the absence of any commercial or financial relationships that could be construed as a potential conflict of interest.

## Publisher's Note

All claims expressed in this article are solely those of the authors and do not necessarily represent those of their affiliated organizations, or those of the publisher, the editors and the reviewers. Any product that may be evaluated in this article, or claim that may be made by its manufacturer, is not guaranteed or endorsed by the publisher.
